# The Priming Effect of Auxiliary Line Construction on Mathematical Creative Thinking: An fNIRS Study

**DOI:** 10.3390/jintelligence14030040

**Published:** 2026-03-03

**Authors:** Chunli Zhang, Kai An, Jiacheng Li, Qinchen Yang, Meihui Song, Li Wang

**Affiliations:** 1 Faculty of Education, Beijing Normal University, Beijing 100875, China; 97070@bnu.edu.cn (C.Z.); 202331010008@mail.bnu.edu.cn (K.A.); 202531010009@mail.bnu.edu.cn (J.L.); 202431010008@mail.bnu.edu.cn (Q.Y.); 202531010010@mail.bnu.edu.cn (M.S.); 2 Beijing Dongcheng District Academy of Educational Science, Beijing 100010, China

**Keywords:** auxiliary line construction, mathematical creative thinking, priming effect, functional near-infrared spectroscopy (fNIRS)

## Abstract

Auxiliary line construction has been identified as a crucial approach to fostering mathematical creative thinking. However, existing studies have only focused on the correlations between auxiliary line construction tasks and mathematical creative thinking, without investigating whether engaging in auxiliary line construction can improve mathematical creativity. As a well-established research paradigm, cognitive priming can elicit changes in thinking within a short period. Based on this idea, the present study adopted the cognitive priming paradigm combined with functional near-infrared spectroscopy (fNIRS) technology, and randomly assigned 42 Chinese college students to an auxiliary line group or a control group. The students’ brain activity was monitored in real time during the priming phase (the auxiliary line group completed geometric problems requiring auxiliary line construction, while the control group finished proof problems with pre-set auxiliary lines) and the post-test phase (both groups completed a mathematical creative thinking test). The behavioral results showed that the auxiliary line group achieved significantly higher scores in fluency and originality of mathematical creative thinking than the control group in the post-test phase. The fNIRS data revealed that during the priming phase, the auxiliary line group exhibited stronger activation of the right superior frontal gyrus and higher variability in dynamic functional connectivity; meanwhile, in the post-test phase, the right superior frontal gyrus and right middle frontal gyrus maintained robust neural activation, and brain functional connectivity exhibited a lower clustering coefficient and attenuated small-world network properties. This study confirms that short-term engagement in auxiliary line construction exerts a priming effect on the fluency and originality of mathematical creative thinking, which may be associated with the enhanced activation of specific brain regions and the dynamic adjustment of brain functional connectivity. These findings provide theoretical and empirical evidence for the cultivation of mathematical creative thinking.

## 1. Introduction

Creative thinking has been widely recognized as a core competency in global education, with the PISA employing “creative thinking” as an assessment indicator (OECD, 2024). As a form of mental exercise, one of the basic aims of mathematics is to generate new ideas and creative solutions to new problems (Mann, 2006). From this perspective, creative thinking is an inherent component of mathematics.

Within mathematics, geometry is a critical factor in fostering creative thinking, and constructing auxiliary lines in geometric problem-solving is particularly closely linked to mathematical creativity (De Vink et al., 2023). In the problem-solving process, auxiliary elements are components conducive to solving geometric problems (Polya, 2004). However, merely obtaining a perceptual understanding of geometric figures (such as identifying figure components) cannot reliably predict mathematical creativity variables; only an operational understanding of geometric figures (such as constructing auxiliary lines) can positively predict the performance of variables in mathematical creative thinking (Gridos et al., 2021; Duval, 1995, 2017). Therefore, the ability to construct auxiliary lines is an important skill for cultivating students’ mathematical creativity.

Existing studies have confirmed a positive correlation between auxiliary line construction and mathematical creative thinking at the behavioral level (Gridos et al., 2021; Muzaini et al., 2023). However, it remains unclear whether engaging in auxiliary line construction can improve mathematical creativity, and neural evidence supporting such a relationship is lacking. From the perspective of cognitive processing, auxiliary line construction can activate spatial dynamic ability (Adiastuty et al., 2020, 2021; Tam & Chan, 2022; Wang et al., 2022), which serves as the core foundation of mathematical creative thinking (Adiastuty et al., 2020, 2021; Palatnik & Sigler, 2019), and may therefore enhance mathematical creative thinking. The present study aims to verify this effect at both the behavioral and neural levels. Functional near-infrared spectroscopy (fNIRS) technology can monitor changes in cerebral blood oxygen levels by leveraging the near-infrared light absorption characteristics of hemoglobin (Naseer & Hong, 2015), thereby reflecting the neural activity of specific brain regions. Thus, it can provide neurological evidence for the effect of auxiliary line construction on mathematical creative thinking.

To explore this research question, the present study adopts the cognitive priming paradigm combined with fNIRS technology. By monitoring participants’ brain activity during the priming and post-test phases, the study investigates the effect of auxiliary line construction on mathematical creative thinking at both the behavioral and neural levels.

## 2. Literature Review

### 2.1. Mathematical Creative Thinking

Mathematical creative thinking is a domain-specific ability to generate unconventional, novel, and effective ideas in mathematical problem-solving and proposition (Leikin, 2009; Suherman & Vidákovich, 2022). Its core dimensions—fluency (number of valid ideas), flexibility (strategy switching), and originality (deviation from conventions)—are tailored to mathematical logic, requiring adherence to rules while diverging from conventional approaches (Pehkonen, 1997).

At the brain region level, this ability relies on specialized functions of key regions within the frontoparietal network: the superior frontal gyrus maintains working memory and exerts executive control over multi-path ideas, the middle frontal gyrus drives idea generation and monitoring of mental simulation, and the inferior parietal lobule mediates logical reasoning and symbol–figure integration (Beaty et al., 2025; Li & Kim, 2025; Skau et al., 2022). These regions work in tandem to balance rigorous verification and the generation of novel ideas, a hallmark of mathematical creativity.

At the brain network level, mathematical creative thinking depends on two core network dynamics: the dynamic balance between the executive control network (goal orientation and idea screening) and the default network (association generation and distant-concept connection), as well as the functional integration of the frontoparietal network (Beaty et al., 2016). Unlike traditional “small-world networks” with high local clustering, creative thinking favors a global integration pattern—characterized by reduced clustering and small-world coefficients—that facilitates cross-module information exchange and distant-concept connections (Savic et al., 2015; Sporns, 2013), laying the neural groundwork for novel mathematical insights.

### 2.2. Auxiliary Line Construction and Its Potential Link to Mathematical Creative Thinking

Auxiliary line construction is a core cognitive operation in geometric problem-solving, defined as modifying figure structures and strengthening hidden relationships to transform unknown problems into known ones (Fan et al., 2017). Its core psychological process centers on breaking conventional cognitive rules of given figures and exploring diverse strategies (e.g., “drawing heights” or “complementing figures”) to reveal hidden connections (Leikin, 2009). This process requires mental simulation of how auxiliary lines alter geometric relationships, activating spatial dynamic ability—a foundational cognitive resource for mathematical creative thinking (Tam & Chan, 2022).

The potential link between auxiliary line construction and mathematical creative thinking stems from overlapping cognitive and neural foundations. Cognitively, auxiliary line construction’s emphasis on strategy exploration directly supports fluency in mathematical creative thinking (efficient generation of multiple ideas), while its perspective-breaking nature nurtures originality (deviation from conventional paths). Though its geometry-oriented focus may not strongly boost flexibility (strategy switching across non-geometric frameworks), its core cognitive processes align with the most critical dimensions of mathematical creative thinking (Gridos et al., 2021).

Neurally, the overlap is evident at both the brain-region and network levels. At the brain-region level, auxiliary line construction enhances activation in the superior frontal gyrus (Skau et al., 2022)—a region critical for proactive control and multi-path idea maintenance in mathematical creative thinking (Li & Kim, 2025)—forming a direct neural basis for their connection. At the network level, auxiliary line construction specifically, as a strategy that imposes additional planning and visuospatial integration demands, may engage frontoparietal control networks. Prior research demonstrates that during abstract reasoning, frontoparietal systems exhibit increased activation and dynamic functional reconfiguration, suggesting their critical role in goal-directed problem solving (Morin et al., 2023), and induces flexibility in dynamic connectivity (frequent switching between information integration modes) (Beaty et al., 2025)—both meeting the network demands of mathematical creative thinking.

Existing correlational evidence supports this multi-level link: Gridos et al. (2021) found that auxiliary line construction ability correlated significantly with fluency (r = 0.38, *p* < 0.01), flexibility (r = 0.36, *p* < 0.01), and originality (r = 0.35, *p* < 0.01) among 243 tenth-grade students, with operational understanding of geometric figures independently predicting these creative dimensions. Muzaini et al. (2023) reported a strong correlation between perspective-breaking via auxiliary lines and creative originality (r = 0.41, *p* < 0.01), a link not fully mediated by spatial visual ability.

### 2.3. Current Study

Existing studies have confirmed a positive correlation between auxiliary line construction and mathematical creative thinking, and have preliminarily indicated that they share an overlapping neural basis (e.g., both involving the frontoparietal network). However, two key research gaps remain unaddressed: firstly, all extant studies focus solely on the static ability level, failing to reveal the relationship between auxiliary line construction and mathematical creative thinking from the perspective of dynamic cognitive processing (e.g., Gridos et al., 2021; Muzaini et al., 2023), or adopt non-randomized intervention approaches (e.g., Fan et al., 2017; Elgrably & Leikin, 2021). Such studies have neither clarified whether auxiliary line construction itself affects mathematical creative thinking nor distinguished its independent effects from the incidental accumulation of geometric knowledge during long-term training. Secondly, the underlying neural correlates between auxiliary line construction and mathematical creative thinking have not been fully explored, as no studies have investigated changes in brain activity during auxiliary line construction—a limitation that hinders an in-depth understanding of their intrinsic neural connections.

To fill these research gaps, the present study aims to address two core scientific questions: Can the short-term process of auxiliary line construction exert a priming effect on mathematical creative thinking? What is the underlying neural basis of this effect?

To achieve this goal, we adopted a single-factor pre-test–post-test experimental design, with Chinese college students randomly assigned to an experimental group (auxiliary line group) and a control group to eliminate baseline differences. The study procedure included three stages: pre-test (assessing mathematical knowledge and general creativity to ensure group homogeneity), priming stage (the auxiliary line group completed geometric problems requiring active auxiliary line construction, while the control group completed proof problems with pre-provided auxiliary lines), and post-test (a mathematical creative thinking test to measure changes in creative performance). Throughout the experiment, functional near-infrared spectroscopy (fNIRS) was used to monitor changes in cerebral blood oxygen levels, enabling real-time tracking of participants’ brain activity during both the priming and post-test stages. Based on the aforementioned theoretical framework, the following two hypotheses are proposed:

(1) Behavioral hypothesis: The auxiliary line group will score significantly higher in mathematical creative thinking in the post-test than the control group, confirming the immediate priming effect.
(2) Neural hypothesis: During the priming stage, the auxiliary line group will show stronger activation in the prefrontal cortex (e.g., superior frontal gyrus, middle frontal gyrus) and higher variability in dynamic functional connectivity; during the post-test stage, they will maintain enhanced activation in these core brain regions and exhibit a brain network pattern with better global integration.

## 3. Materials and Methods

### 3.1. Participants

The study initially recruited 45 undergraduate and graduate students. One participant was excluded due to a score of 0 on the mathematical knowledge test, indicating a lack of foundational knowledge for subsequent problem-solving. During the fNIRS data quality analysis, two participants were excluded because their number of bad channels exceeded 30%. Consequently, the final effective sample consisted of 42 participants (33 female), with an average age of 22.78 years. These participants were randomly assigned to two groups of 21 each. All participants were native Chinese speakers, had normal or corrected-to-normal vision, and were not color-blind or color-weak. All participants were from universities in Beijing and had not previously participated in similar experiments. This study was approved by the Research Ethics Review Committee of the Faculty of Education at Beijing Normal University (BNU202201100003). All participants signed an informed consent form before the experiment and received compensation upon completion.

### 3.2. Experimental Design

**Figure 1:**
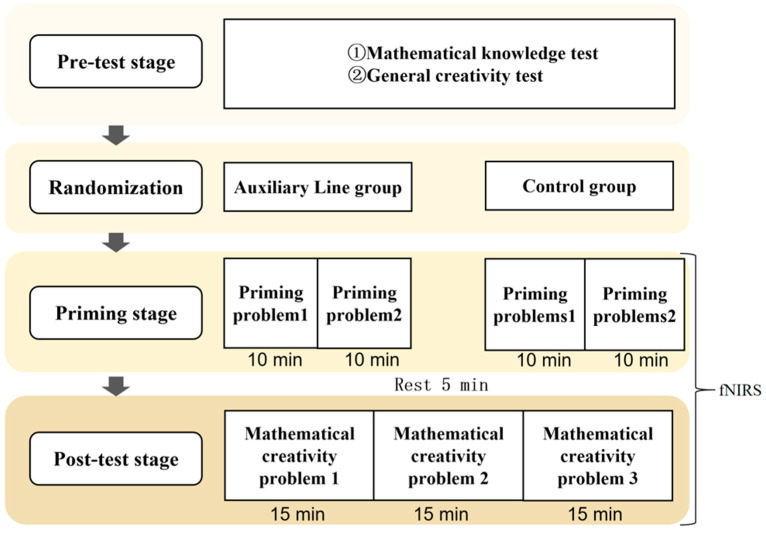
Procedure of experiment.

This study employed a single-factor pre–post-test design. The procedure was as follows (Figure 1): first, a pre-test was administered to the initially recruited 45 participants, covering mathematical knowledge and general creativity. After excluding one unsuitable participant, the remaining participants were randomly assigned to two groups of 22 each (two participants were later excluded during data analysis, resulting in a final sample of 21 per group). Statistical tests confirmed no significant differences in pre-test scores between the two groups. At least one week after the pre-test, participants took part in the fNIRS experiment, which lasted approximately 1.5 h. In the priming stage, the auxiliary line group completed two priming problems requiring the construction of auxiliary lines within 20 min. To match this duration, the control group completed four proof-only problems. Then, all participants rested for 5 min, immediately followed by a post-test of mathematical creative problem-solving, where all participants were given 15 min to solve three mathematical creativity problems, totaling 45 min.

### 3.3. Tests

#### 3.3.1. Pre-Tests

Mathematical knowledge test: The mathematical knowledge test was designed to assess the participants’ understanding of the concepts related to the mathematical problems presented in both the priming stage and the mathematical creativity post-test. It assessed knowledge of the area of a trapezoid and proofs involving triangles. The test consisted of 2 items, each with a maximum score of 10 points, for a total of 20 points. The specific items are listed in Appendix A.

General creativity test: This task employed the Alternative Uses Test. Participants were instructed to imagine a piece of glass and generate as many uses for it in daily life as possible. There were no limitations on the size or quantity of the glass. The instructions emphasized that “the more uses, the better” and “the more unique, the better,” encouraging participants to think of applications that others might not. The responses were scored on three dimensions: fluency, flexibility, and originality.

#### 3.3.2. Priming Tests

Auxiliary line group: Participants solved two geometry proof problems, with the explicit instruction to use auxiliary lines. They were required to write down only the key steps and were encouraged to find multiple solutions for each problem, with a 10 min time limit per problem (see Appendix B).

Control group: Participants solved four geometry proof problems where the auxiliary lines were already provided. They were instructed to write out the proof in as much detail as possible. Due to the lower difficulty of this task, participants completed two problems within each 10 min block (see Appendix B).

#### 3.3.3. Post-Tests

All participants completed three mathematical creativity tasks (see Appendix C). Task 1 was the graph-drawing task developed by Burger and Shaughnessy (1986), and Task 2 was the 24-point task developed by Leikin and Lev (2007); both are applicable for measuring mathematical creative thinking. Task 3 required students to explore multiple solutions to a geometric proof problem, similarly to the mathematical creative thinking task developed by Leikin (2009). To facilitate subsequent data processing, participants would raise their hand to indicate when they had completed an answer, and the experimenter would make a note during the fNIRS data collection process. The behavioral indicators for all tasks were scored based on three dimensions: fluency, flexibility, and originality (as shown in Appendix D).

### 3.4. fNIRS Data Acquisition

**Figure 2:**
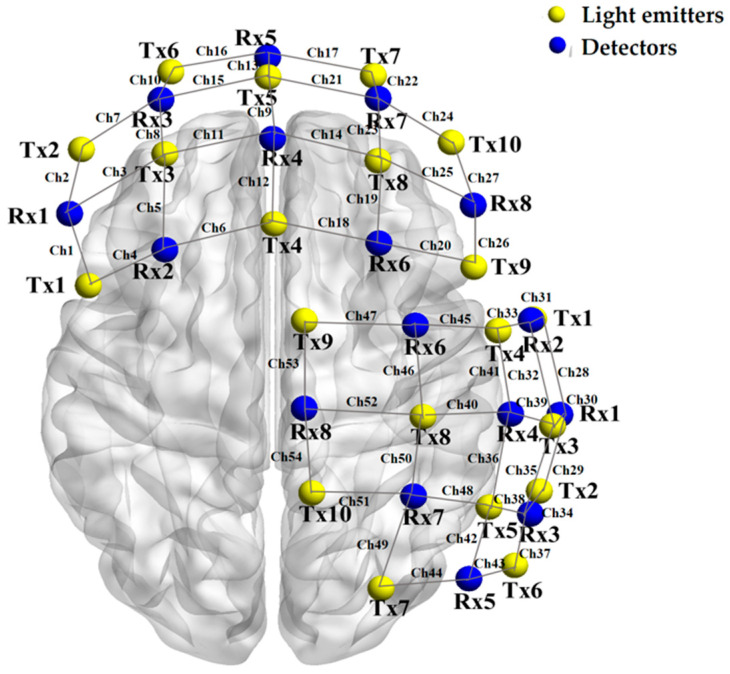
Location of the near-infrared photoelectrode and channels.

Data were collected using a Brite MK III wireless, wearable fNIRS system (Artinis Medical Systems, Elst, Gelderland, The Netherlands) with a sampling rate of 25 Hz and wavelengths of 760 nm and 850 nm. A dual-Brite setup was used, with each device containing 10 light emitters and 8 detectors, forming 27 channels with an optode distance of 3 cm. In total, 54 channels were recorded. Previous studies have found that the regions activated by mathematical processing are wide-ranging and dominated by the frontal parietal network (Beaty et al., 2016; Flaherty, 2005), and neuroscience research often links creativity with activities in the right hemisphere (Bogen, 1969). Therefore, following the international 10–20 system, Device 1 covered the prefrontal cortex, and Device 2 covered the right parietal and temporal lobes, as shown in Figure 2 and Appendix E.

### 3.5. Data Analysis

#### 3.5.1. Behavioral Data Analysis

Creativity was evaluated across three dimensions: fluency, flexibility, and originality. Responses were scored by two trained raters following a standardized calibration protocol, who achieved high inter-rater reliability (intra-class correlation coefficients > 0.87). Independent samples *t*-tests were subsequently conducted to assess inter-group differences in behavioral outcomes. Details of the scoring criteria and calculation methods are provided in Appendix F.

#### 3.5.2. fNIRS Data Analysis

fNIRS data processing was performed using the Homer3 (v1.80.2) toolbox. Preprocessing involved a rigorous quality control pipeline, including artifact rejection, wavelet-based motion correction, PCA denoising, and bandpass filtering to isolate hemodynamic signals and extract oxygenated hemoglobin (HbO) concentrations. For the priming stage, we analyzed the temporal evolution of brain activity using a time-segmented General Linear Model (GLM) and examined network stability and state dynamics through dynamic functional connectivity (dFC). For the post-test stage, a trial-by-trial GLM was constructed to estimate activation for unique solutions; furthermore, Partial Least Squares Correlation (PLSC) and graph-theoretical approaches were employed to explore multivariate brain–behavior associations and network topological properties (e.g., small-worldness). Details of the preprocessing parameters, mathematical definitions of network metrics, and full statistical procedures are given in Appendix F.

## 4. Results

### 4.1. Behavioral Results

Descriptive statistics and independent samples *t*-tests were conducted on the behavioral data. As shown in Table 1, there were no significant differences in mathematical knowledge or in the three dimensions of general creativity (all *p* > 0.05) between the two groups during the pre-test stage, indicating that the groups were homogeneous at baseline.

During the priming stage, there were no significant differences in behavioral scores between the groups (t = 0.12, *p* = 0.91), suggesting that the priming tasks were of comparable difficulty.

In the post-test of mathematical creativity, the auxiliary line group scored significantly higher in fluency (t = 2.06, *p* = 0.04, d = 0.63) and originality (t = 2.08, *p* = 0.04, d = 0.64).

### 4.2. Priming Stage fNIRS Results

#### 4.2.1. Activation Analysis Results of Priming Stage

**Figure 3:**
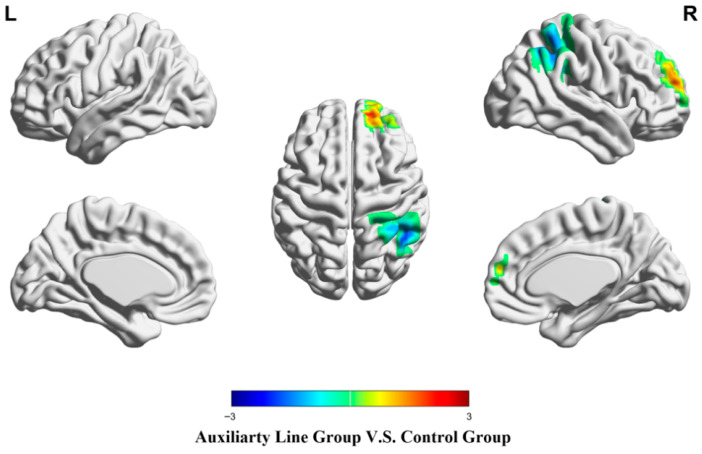
Brain activation differences during the priming stage.

During the priming stage, the auxiliary line group showed significantly stronger activation in the right superior frontal gyrus than the control group (*p* < 0.05). Conversely, the control group exhibited stronger activation in a part of the right inferior parietal lobule (*p* < 0.05), as shown in Figure 3 and Table 2.

#### 4.2.2. Brain–Behavior Correlation Analysis Results

**Figure 4:**
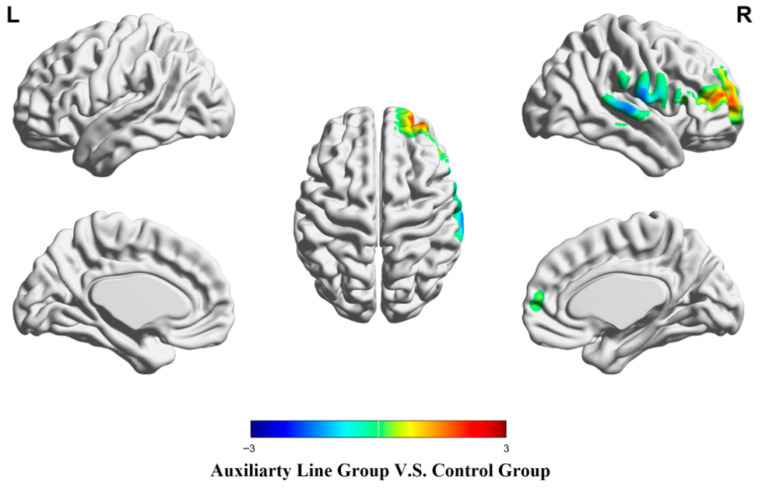
Differences in brain behavior correlations during the priming stage.

A comparison of Fisher’s Z-transformed brain–behavior correlations between the groups revealed that the correlation was significantly greater for the auxiliary line group in the right superior frontal gyrus (*p* < .05). In contrast, the correlation was significantly greater for the control group in the right superior temporal gyrus (*p* < .05; see Figure 4 and Table 3). This suggests that in the auxiliary line group, performance was a better predictor of activation in the right superior frontal gyrus, whereas in the control group, performance was a better predictor of activation in the right superior temporal gyrus.

#### 4.2.3. Results of Dynamic Functional Connectivity Analysis

The analysis of the activation time course revealed significant inter-group differences at multiple time points (*p* < .05), as shown in Appendix G.

**Figure 5:**
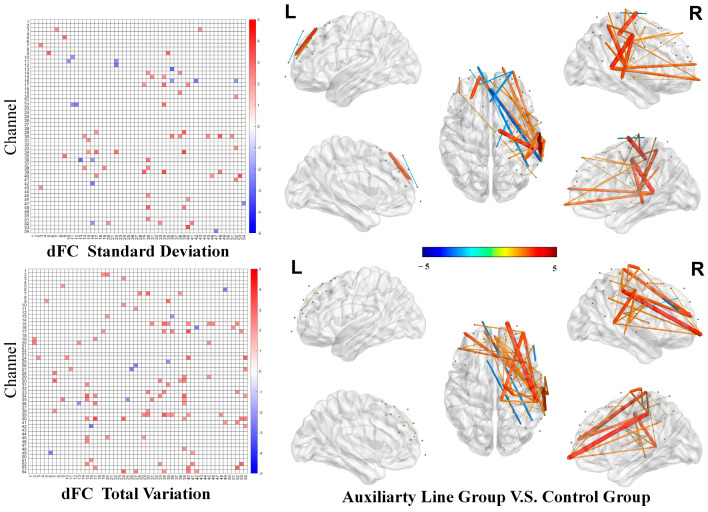
Differences in dynamic functional connectivity analysis of variability during the priming stage.

In addition, the variability analysis of dynamic functional connectivity showed that the auxiliary line group demonstrated significantly greater variability in dynamic functional connectivity compared to the control group. Specifically, the auxiliary line group showed greater standard deviation in connectivity between multiple brain regions, as evidenced by higher variability (76% of significant connections; see top of Figure 5) and greater total variation (90% of significant connections; see bottom of Figure 5). This indicates a more flexible pattern of brain information exchange in the auxiliary line group during the priming stage.

**Figure 6:**
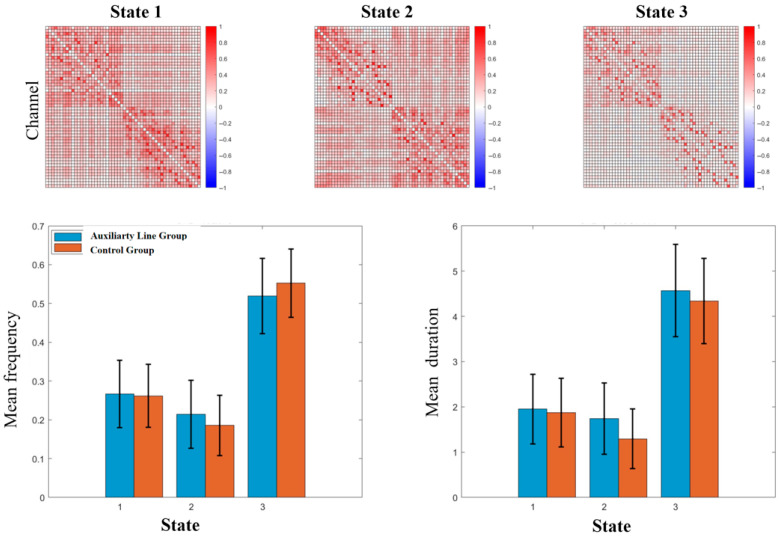
Differences in state analysis of dynamic functional connectivity during the priming stage.

Finally, the analysis of dynamic functional connectivity identified three distinct brain functional connectivity states, and there were no significant inter-group differences in the frequency or duration of any state (*p* > 0.05) (see Figure 6). However, a significant difference was found in the transition pattern between states, with the auxiliary line group presenting a significantly higher probability of transitioning from State 2 to State 1 (19.54%) compared to the control group (4.83%) (*p* = 0.02).

### 4.3. Post-Test Stage fNIRS Results

#### 4.3.1. Activation Analysis Results of Post-Test Stage

**Figure 7:**
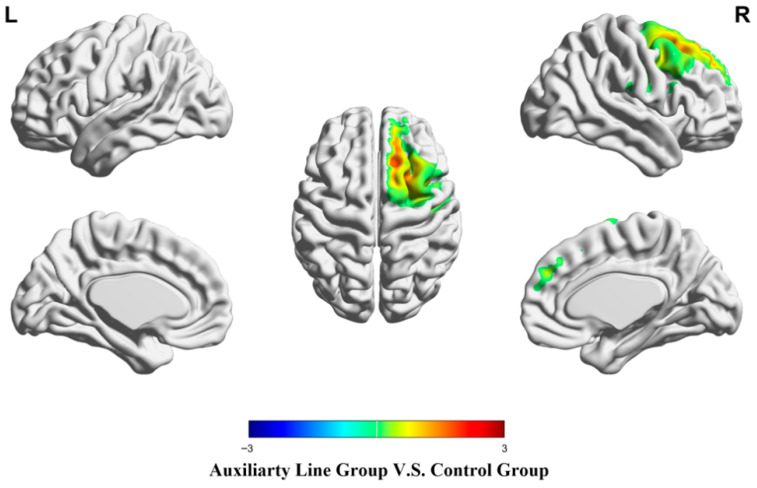
Brain activation differences during the post-test stage.

During the post-test of mathematical creativity, the auxiliary line group showed significantly stronger activation in the right superior frontal gyrus than the control group (*p* < .05), while there were no regions where the control group showed stronger activation than the auxiliary line group (*p* > .05) (see Figure 7 and Table 4).

#### 4.3.2. Brain Behavior Correlation Analysis Results

**Figure 8:**
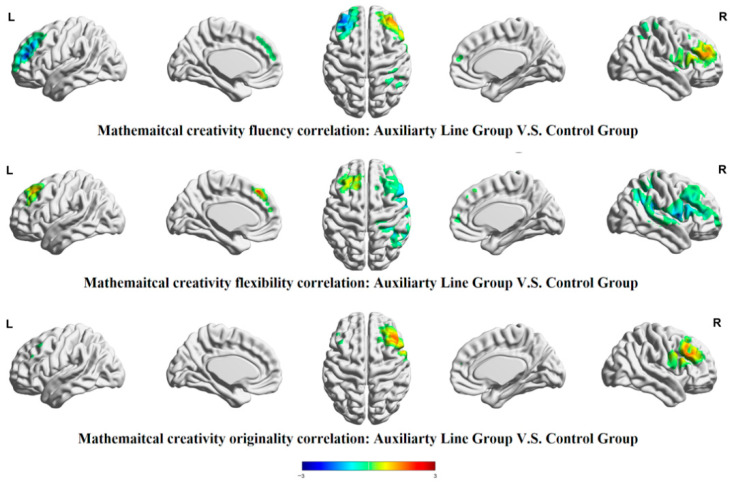
Differences in brain behavior correlations for each mathematical creative thinking dimension during the post-test stage.

Group comparisons of brain behavior correlations revealed distinct patterns for each dimension of mathematical creative thinking, as shown in Figure 8 and Table 5: in the fluency dimension, the auxiliary line group showed a stronger positive correlation in the right middle frontal gyrus and a stronger negative correlation in the left middle frontal gyrus and right superior parietal lobule than the control group (*p* < .05). In the flexibility dimension, the auxiliary line group showed a stronger correlation in the left medial superior frontal gyrus and a stronger negative correlation in the right superior temporal gyrus, right middle temporal gyrus, and right angular gyrus than the control group (*p* < .05). In the originality dimension, the auxiliary line group showed a stronger correlation in the right middle frontal gyrus and a stronger negative correlation in the left middle frontal gyrus than the control group (*p* < .05). Overall, compared to the control group, the auxiliary line group more effectively recruited the right middle frontal gyrus when working on tasks measuring fluency and originality of mathematical creative thinking, and the left medial middle frontal gyrus when working on tasks measuring flexibility, allowing them to achieve higher scores.

Further, we conducted a Partial Least Squares Correlation (PLSC) analysis to comprehensively investigate the differences in brain behavior correlation patterns between the two groups, as shown in Appendix H, and the results are consistent with our other findings.

#### 4.3.3. Functional Connectivity Analysis Results

**Figure 9:**
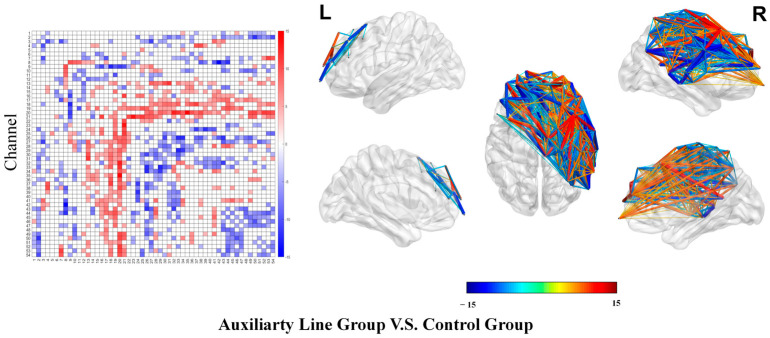
Differential in functional connectivity during the post-test stage.

In the functional connectivity analysis, both groups showed significant correlations across all channels, and there were 448 channel pairs with significantly different correlations between the two groups (*p* < 0.05; see Figure 9).

**Figure 10:**
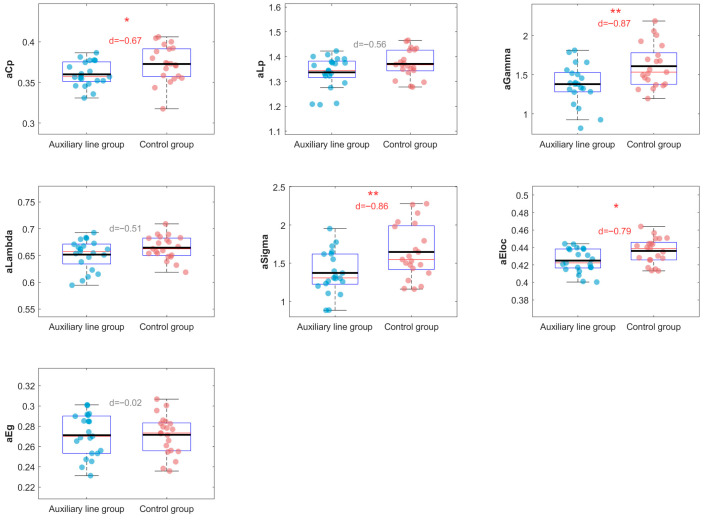
Difference in graph theory metrics of functional connectivity during the post-test mathematical creative thinking stage. *, *p* < 0.05. **, *p* < 0.01.

Further, the results of the analysis of graph theory metrics for functional connectivity are shown in Figure 10; they reveal significant differences in the clustering coefficient (aCp) and the normalized clustering coefficient (aGamma) (*p* < 0.05), indicating a higher degree of functional segregation in the control group’s brain network. A significant difference was also found in the small-world coefficient (aSigma) (*p* = 0.012, d = −0.82), suggesting stronger small-world properties in the brain networks of participants in the control group. Furthermore, the control group exhibited stronger local efficiency (aEloc) (*p* = 0.015, d = −0.79), indicating more efficient local communication within their brain networks. No significant inter-group differences were observed for the other metrics.

#### 4.3.4. Supplementary Analysis

To further investigate the predictive effect of the priming stage on the post-test mathematical creative thinking stage, we conducted a correlation analysis of the brain data from the priming stage and the brain data for mathematical creative thinking for both groups, as shown in Appendix I.

## 5. Discussion

Adopting a cognitive priming paradigm combined with fNIRS, this study confirms behaviorally and neurally that short-term auxiliary line construction significantly promotes the fluency and originality of mathematical creative thinking. This effect relies on altered activation patterns in specific brain regions and the dynamic reconstruction of brain network topology.

### 5.1. Behavioral Promoting Effect of Auxiliary Line Construction on Mathematical Creative Thinking

The 20 min auxiliary line construction task exerted an immediate priming effect on mathematical creative thinking fluency (t = 2.06, *p* = 0.04, d = 0.63) and originality (t = 2.08, *p* = 0.03, d = 0.64). Existing studies have confirmed that auxiliary line construction, as the core operation of solving geometric problems, is closely related to mathematical problem-solving (e.g., Palatnik & Dreyfus, 2019). Some studies have attempted to improve the ability to use auxiliary lines through long-term geometric training, with the aim of improving mathematical performance (e.g., Fan et al., 2017; Elgrably & Leikin, 2021). However, its effect is often impacted by factors such as training duration and task complexity, and it is difficult to distinguish the independent effects of “the auxiliary line construction operation itself” and “the accompanying accumulation of spatial knowledge” (Hsu & Silver, 2014; Lev & Leikin, 2013). This study adopted a 20 min auxiliary line construction task (actively generating and simulating multiple auxiliary lines and evaluating their value), which effectively avoided the interference of “knowledge accumulation” in long-term training. This is consistent with the research results of Singer and Voica (2015), whose short-term modeling training (similar to structured cognitive operations) was found to improve mathematical creative thinking scores by 22% (d = 0.61), with a similar effect size.

This behavioral effect may stem from auxiliary line construction’s role in priming the cognitive framework via two key operations: internal mental simulation and perspective switching. First, simulating geometric structural changes engages internal dynamic spatial ability, a core foundation of mathematical creativity (Adiastuty et al., 2020, 2021; Leikin, 2009). Second, perspective switching breaks initial figure cognition to explore hidden relationships, aligning with external dynamic spatial ability and the creative need to transcend conventional frameworks (Tam & Chan, 2022; Adiastuty et al., 2020, 2021; Palatnik & Sigler, 2019). These operations precisely target the demands of fluency (idea generation) and originality (perspective shifting) (Muzaini et al., 2023; Gridos et al., 2021; Mann, 2006). This is supported by findings that efficient mental simulation correlates with higher fluency (Leikin, 2009), while strong perspective-breaking ability is linked to higher originality (Palatnik & Sigler, 2019). Additionally, cognitive processes like problem representation and strategy retrieval may also contribute to achieving such results (Gridos et al., 2021).

The lack of significant improvement in flexibility may be attributed to the “geometry-oriented” nature of auxiliary line construction. By reinforcing a single cognitive path of “geometric operation,” the task likely induces a temporary “thinking set” that increases the cognitive cost of switching between diverse problem-solving frameworks (e.g., algebraic, logical) (Palatnik & Sigler, 2019). This aligns with the results of Skau et al. (2022), whose comparison between text- and geometry-based tasks showed that students who focus on geometry for an extended period have a 20% longer reaction time when switching to algebraic tasks.

### 5.2. Brain Region Activation and Network Flexibility During the Priming Stage

The fNIRS results from the priming stage reveal neural differences between the experimental and control groups, with the auxiliary line group showing stronger activation in the right superior frontal gyrus, and the control group in the right inferior parietal lobule. The fNIRS results during the priming stage reveal potential neural differences associated with auxiliary line construction: activation analysis showed that the auxiliary line group exhibited significantly stronger activation in the right superior frontal gyrus than the control group (t = 3.12), while the control group showed stronger activation in the right inferior parietal lobule (t = −2.52). This difference may reflect differential engagement of multiple brain regions: the “multi-path generation and evaluation” of auxiliary line construction may involve increased engagement of the right superior frontal gyrus, in order to monitor the rationality of auxiliary lines, and adjust the direction of mental simulation (Palatnik & Dreyfus, 2019; Li & Kim, 2025); this is consistent with the research results of Leikin et al. (2011), who found that mathematically gifted students have 28% stronger activation in the superior frontal gyrus in multi-path geometric tasks. The control group’s “proving based on given auxiliary lines” focuses on logical reasoning, thus activating the right inferior parietal lobule, which is involved in symbol processing and logical operations (Herbst & Brach, 2006; Palatnik & Dreyfus, 2019; Skau et al., 2022). Skau et al. (2022) further verified that the activation of the inferior parietal lobule is positively correlated with reasoning accuracy (r = 0.35, *p* < 0.05). This comparison indicates that auxiliary line construction may be associated with a more exploratory cognitive state, rather than mere activation of mathematical knowledge (Beaty et al., 2016).

Dynamic functional connectivity analysis further shows that auxiliary line construction may be associated with changes in brain network properties by enhancing network fluidity and state transition probability. The results of this study showed that although both groups of participants had three similar brain network states, the transition probability between states in the auxiliary line group (especially the transition from state 2 to state 1, with 19.54% in the auxiliary line group vs. 4.83% in the control group) was significantly higher, and it showed stronger connection variability in 76% of the significantly activated channels and greater total variation in 90% of the channels. This pattern suggests that auxiliary line construction may help the brain network to present higher “fluidity”, in other words, more frequent switching between different information integration modes (Muzaini et al., 2023; Gridos et al., 2021; Beaty et al., 2025). This is broadly consistent with the findings of Beaty et al. (2025), who found that highly creative individuals have a 70% higher network switching frequency, and short-term training can enhance this ability.

The enhanced brain network flexibility induced by auxiliary line construction is consistent with the potential neural correlates of mathematical creative thinking, which relies on flexible concept connection and switching between divergent and convergent thinking. Neuroscientific research on creativity points out that the core of mathematical creative thinking lies in flexibly connecting distant concepts and freely switching between divergent exploration and convergent screening, and a dynamically flexible brain network may represent an important neural correlate for realizing this cognitive process (Dietrich & Kanso, 2010; Beaty et al., 2016; Bassett & Bullmore, 2006; Leikin et al., 2011). Through “multi-path exploration”, auxiliary line construction can essentially “warm up” the dynamic conversion ability of the brain network, potentially providing a neural context for subsequent creative tasks; Leikin et al. (2011)’s longitudinal study confirmed that continuous multi-path training can improve network conversion efficiency by 30%, and the stability lasts for more than 3 months.

### 5.3. Frontoparietal Network Activity and Global Integration Mode During the Post-Test Stage

The cognitive priming effect of auxiliary line construction was found to transfer to the post-test stage, with the auxiliary line group maintaining strong activation in the right superior frontal gyrus and middle frontal gyrus, which correlates closely with fluency and originality in mathematical creative thinking. This behavioral phenomenon is clearly reflected at the neural level: activation analysis showed that the auxiliary line group still maintained strong activation in the right superior frontal gyrus during the post-test (t = 2.26; t = 2.15), and the positive correlation between the activation of this brain region and the fluency and originality of mathematical creative thinking was significantly stronger than that in the control group. This indicates that an “exploratory executive control mode” in the context of the present task appeared to be maintained with the end of the priming task, but was effectively applied to the new creative thinking process, suggesting that continuous participation of the prefrontal cortex in “idea generation and monitoring” may support fluency and originality of mathematical creative thinking (Beaty et al., 2016; Benedek et al., 2014; Li & Kim, 2025); Li and Kim (2025)’s fMRI meta-analysis also found that the average correlation coefficient between the activation of the superior frontal gyrus and creative scores was 0.38 (*p* < 0.01).

Brain–behavior correlation and predictive analysis indicate that auxiliary line construction may preferentially engage frontoparietal networks, which support advanced problem-solving and creative thinking. Further brain–behavior correlation analysis showed that the activation of the right middle frontal gyrus in the auxiliary line group was more strongly correlated with fluency and originality, and the activation intensity of the right superior frontal gyrus and middle frontal gyrus during the priming stage could significantly predict the level of creative-brain activation during the post-test stage. This result indicates that auxiliary line construction may not uniformly activate the brain network, but specifically strengthens the neural pathway of the “frontoparietal network” which is closely related to advanced problem-solving. Through “multi-path exploration” in auxiliary line construction, the functional connection and activation patterns may be temporarily modulated, and could thus show stronger participation in subsequent creative tasks (Beaty et al., 2016; Benedek et al., 2014; Zhu et al., 2017); Creative performance has been associated with altered functional connectivity involving the frontoparietal network, characterized by reduced within-network connectivity but increased coupling between the frontoparietal and default mode networks. Notably, these effects appear particularly robust for figural creativity (Zhu et al., 2017). 

The exploration of graph theory analysis suggests that mathematical creative thinking involves a globally distributed network configuration. Although the auxiliary line group demonstrated better creative performance, it exhibited significantly lower normalized clustering (1.37 vs. 1.55, *p* = 0.013, d = −0.80) and a lower small-world coefficient (1.35 vs. 1.55, *p* = 0.012, d = −0.82) compared to the control group. Early studies in network science emphasize that small-world networks—characterized by high local clustering and short global paths—are often considered optimal for efficient information processing (Bassett & Bullmore, 2006; Latora & Marchiori, 2001). However, in the context of creativity, high clustering implies rigid modularity, which hinders novel associations. The observed reduction therefore reflects a strategic shift toward a less segregated, globally integrated topology, facilitating the “distant connections” critical for insight (Beaty et al., 2016). This aligns with the findings of Savic et al. (2015), who found that lower clustering correlated with higher originality in mathematical proofs (r = −0.36, *p* < 0.05). Furthermore, reduced modularization lowers barriers to information flow, fostering cross-domain integration (Sporns, 2013; Bassett & Bullmore, 2006). These findings imply that auxiliary line construction induces temporary reorganization of functional network topology to support creative thinking.

### 5.4. Implications for Education

This study provides targeted and operable practical implications for mathematics education. First, short-term auxiliary line construction (e.g., 20 min multi-solution auxiliary line exercises) can serve as a “cognitive priming tool” in geometric teaching, activating students’ mathematical creative thinking before open-ended problem-solving or design tasks. Second, teachers can incorporate auxiliary line construction into regular lessons to encourage unconventional thinking and creative problem-solving, without the need for additional specialized training. Third, our findings suggest that focusing on mental simulation and perspective switching in geometry teaching, rather than merely emphasizing knowledge and skill mastery, can better promote mathematical creative thinking.

### 5.5. Limitations

Although the cognitive priming paradigm combined with fNIRS provides empirical evidence for the short-term priming effect of auxiliary line construction on mathematical creative thinking and its underlying neural mechanisms, this study still has several limitations. First, the small sample size (42 participants) may limit the generalizability of the findings at the neural level; second, in terms of teaching practicality, the experimental design of this study failed to fully control other confounding variables, leading to alternative explanations for the research findings, which warrant further investigation; and third, the notion that lower clustering density and small-world properties reflect the level of global integration remains controversial at the theoretical level, and should thus be interpreted with caution.

## 6. Conclusions

Using a cognitive priming paradigm combined with fNIRS, this study confirms that a 20 min auxiliary line construction task exerts a significant, stable, short-term enhancing effect on the fluency and originality of mathematical creative thinking, with no significant effect on flexibility. Neurally, the task increased activation in the right superior and middle frontal gyri across both the priming and post-test phases. Furthermore, it heightened the variability in dynamic functional connectivity during priming and induced a globally integrated network topology (characterized by lower clustering and small-world coefficients) in the post-test. These findings elucidate the neural mechanisms underlying this effect and offer a feasible cognitive priming tool for fostering mathematical creativity in education.

## Figures and Tables

**Table 1:** Descriptive statistics and *t*-test results.

Stage	Tests	Group	M (SD)	t	*p*	d
Pre-test	1. Mathematical knowledge test	ALG	14.90 (4.55)	−0.87	0.39	−0.27
		CG	16.19 (4.95)			
	2.1. General creativity fluency	ALG	13.43 (5.60)	0.91	0.37	0.28
		CG	12.10 (3.70)			
	2.2. General creativity flexibility	ALG	11.24 (4.93)	0.52	0.61	0.36
		CG	10.57 (3.20)			
	2.3. General creativity originality	ALG	1.33 (2.54)	−1.10	0.28	−0.34
		CG	2.14 (2.22)			
Priming	3. Priming test	ALG	30.48 (5.22)	0.12	0.91	0.04
		CG	30.29 (5.29)			
Post-test	4.1. Mathematical creative thinking fluency	ALG	51.90 (21.77)	2.06 *	0.04	0.64
		CG	39.62 (16.44)			
	4.2. Mathematical creative thinking flexibility	ALG	22.33 (5.73)	1.18	0.25	0.36
		CG	20.14 (6.31)			
	4.3. Mathematical creative thinking originality	ALG	11.57 (4.35)	2.08 *	0.04	0.64
		CG	9.14 (3.11)			

Note. ALG, auxiliary line group. CG, Control Group. *, *p* < 0.05.

**Table 2:** Coordinates and statistical parametric of brain activation differences during the priming stage.

Channel	Brain Region	x	y	z	t
21	R Superior frontal gyrus	13	59	21	3.12
42	R Inferior Parietal Lobule	50	−57	49	−2.52

**Table 3:** Coordinates and statistical parametric of differences in brain behavior correlations during the priming stage.

Channel	Brain Region	x	y	z	r1	r2	z
23	R Middle Frontal Gyrus	29	60	35	0.43	−0.20	1.99
24	R Middle Frontal Gyrus	38	62	15	0.42	−0.47	2.86
30	R Superior Temporal Gyrus	72	−22	7	−0.42	0.52	−3.09

**Table 4:** Coordinates and statistical parametric of brain activation differences during the post-test stage.

Channel	Brain Region	x	y	z	t
14	R Middle Frontal Gyrus	15	46	53	2.26
18	R Middle Frontal Gyrus	15	24	59	2.15

**Table 5:** Coordinates and statistical parametric of differences in brain behavior correlations for each mathematical creative thinking dimension during the post-test stage.

Behavior Index	Ch.	Brain Region	x	y	z	r1	r2	z
Mathematical creative thinking fluency	2	L Middle Frontal Gyrus	−49	40	22	−0.48	0.34	−2.61
	25	R Middle Frontal Gyrus	42	42	39	0.26	−0.38	2.00
	27	R Middle Frontal Gyrus	52	44	23	0.31	−0.34	2.03
	48	R Superior parietal lobule	44	−47	63	−0.16	0.47	−2.03
Mathematical creative thinking flexibility	12	L Medial superior frontal	−1	30	47	0.51	−0.32	2.75
	31	R Superior Temporal Gyrus	62	3	6	−0.03	0.56	−1.99
	37	R Middle temporal gyrus	62	−54	16	−0.46	0.25	−2.27
	43	R Angular	60	−61	33	−0.34	0.32	−2.06
Mathematical creative thinking originality	3	L Middle Frontal Gyrus	−42	39	44	−0.39	0.29	−2.11
	26	R Middle Frontal Gyrus	54	25	39	0.32	−0.43	2.39

**Figure A1:**
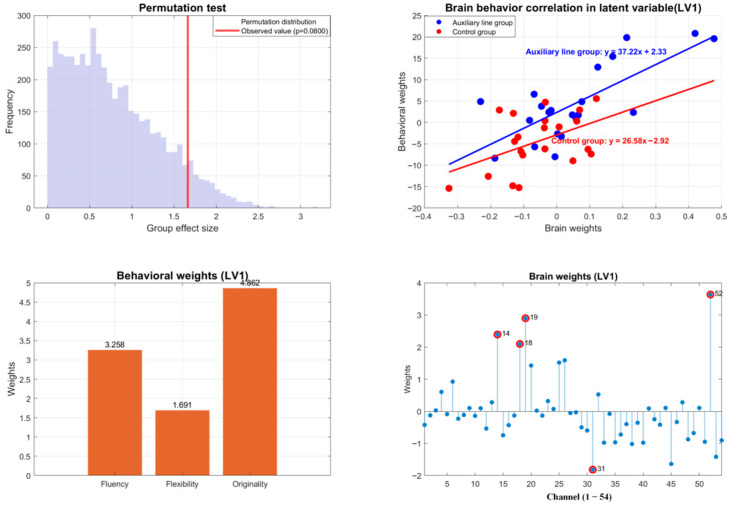
PLSC analysis results of brain-behavior correlation during the post-test stage.

**Figure A2:**
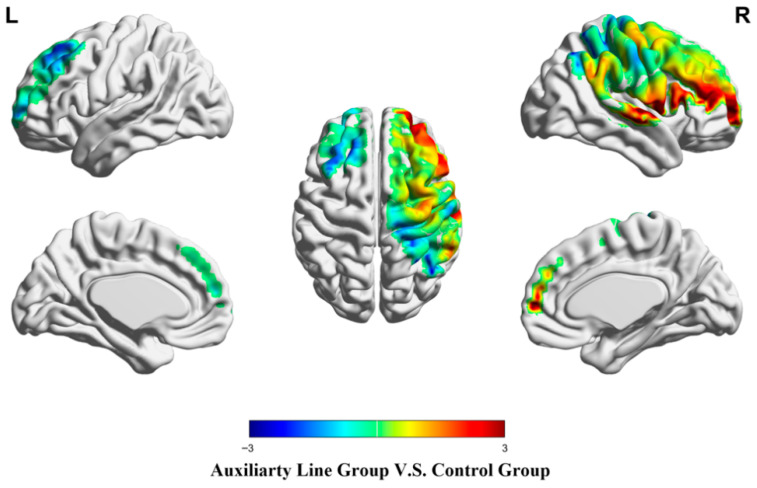
Difference in correlation between priming stage and post-test brain activation.

## Data Availability

The data that support the findings of this study are available on request from the corresponding author, upon reasonable request.

## Appendix A. Mathematics Knowledge Test

1. Please write down the derivation method of the area of a trapezoid.

2. In △ABC, AB = AC, and ⊙O is the circumcircle of △ABC. The extension of BO intersects side AC at point D.
  (1) Prove that: ∠BAC = 2∠ABD;
  (2) When △BCD is an isosceles triangle, find the measure of ∠BCD.

## Appendix B. Tasks for the Priming Stage

### Appendix B.1. Auxiliary Line Group Questionnaire

Note: The following questions are about auxiliary lines. You have 20 min to complete the answers to 2 questions. Please be sure to use the method of introducing auxiliary lines to solve them.

1. Prove that the area of triangle AED is half the area of parallelogram ABCD. (Please prove it in as many ways as possible, and each method must use auxiliary lines. Just write down the key steps. If the reserve figures are not enough, you can add your own. Time limit: 10 min)

2. Please divide the trapezoid into two parts of equal area with a straight line. (Please try to draw the division methods in as many ways as possible. Just write down the key steps. If the backup pictures are not enough, you can add your own. Time limit: 10 min.)

### Appendix B.2. Control Group Questionnaire

Note: The following is a two-page math test. You will have 10 minutes to complete the 2 questions on the first page, followed by another 10 minutes to complete the 2 questions on the second page.

Page 1 content:

1. Prove that the area of triangle AED is half the area of parallelogram ABCD. (Please write down all the steps as detailed as possible.)

2. Given trapezoid ABCD, take point E on AD and point F on BC, and connect EF. Please prove that the area of quadrilateral ABEF is equal to the area of quadrilateral EFDC. (Please write down all the steps as detailed as possible.)

Page 2 content:

1. Prove that the area of triangle AED is half the area of parallelogram ABCD. (Please write down all the steps as detailed as possible.)

2. Given trapezoid ABCD as shown in the figure, E is the midpoint of AB and F is the midpoint of CD. Connect EF. Please prove that the area of quadrilateral ADFE is equal to the area of quadrilateral EFCB. (Please write down all the steps as detailed as possible.)

## Appendix C. Mathematical Creative Thinking Test

1. Draw a polygon with an area of 2 cm^2^ on the dotted paper. Please ensure that the polygons you draw are different (the horizontal and vertical distances between these points are 1 cm). (Please answer in the order of the numbers. Time limit: 15 min.)

For example:

2. Remove the two jokers from a deck of playing cards. From the remaining 52 cards, randomly draw 4 cards to form a hand. Using any arithmetic operations such as addition, subtraction, multiplication, and division (parentheses are allowed), make the final result of the hand equal to 24. Each card in the hand must be used exactly once. (Please write down as many hands and equations as you can think of. Different calculations for the same hand count as two answers. Time limit: 15 min.)

For example, if the card set is 3, 8, 8, 9, the formulas are ① (9 − 8) × 8 × 3, ② (9 − 8 ÷ 8) × 3. Note: (9 − 8) × 8 × 3, (8 − 7) × 8 × 3, and (7 − 6) × 8 × 3, etc., are regarded as the same algorithm and will not be scored repeatedly. (Please mark the answers in the order of answering, such as ①, ②…)

3. AB is the diameter of circle O, and C and D are points on circle O such that AC // OD. E is the intersection point of AC and DB. Prove that DB = DC. (Please provide as many methods of proof as possible. Number your answers in the order you provide them, such as ①, ②, etc. Time limit: 15 min)

## Appendix D. Scoring Criteria for Mathematical Creative Thinking Test

### Appendix D.1. Scoring Criteria for Question 1

(1) Fluency: One point is awarded for each correct answer.
(2) Flexibility: One point is awarded for each category change.

Classification Criteria: Triangles (isosceles triangles, right-angled triangles), regular shapes (squares, rectangles, parallelograms, symmetrical irregular quadrilaterals), irregular shapes (can be decomposed into two or more regular shapes, including: squares, right-angled triangles, isosceles triangles, trapezoids, parallelograms)

Note: Different ways of joining are considered different types (e.g., joining at the corners of two right-angled triangles, joining at a corner and a side, or joining at the sides are all considered different types).

(3) Originality: Originality was evaluated using a statistical infrequency approach based on the distribution of responses across the full sample. Responses occurring in less than 1% of the sample were awarded 5 points; those occurring in 1.01–3% received 4 points; 3.01–5% received 3 points; 5.01–10% received 2 points; 10.01–15% received 1 point; and responses occurring in more than 15% of the sample received 0 points.

### Appendix D.2. Scoring Criteria for Question 2

(1) Fluency: The number of correct answers.
(2) Flexibility: Conversion between algorithms (including +, −, ×, ÷). For example, 5 + 5 + 5 + 9, 5 × 4 + 2 + 2, 3 + 4 + 8 + 9, 4 × 8 − 5 − 3, flexibility scores 3 points.
(3) Originality: Scored criteria same with Question 1.

Note: Simple rearrangement of positions does not count as originality, such as 12 × 4 ÷ 2 ÷ 1 and 12 ÷ 1 × 4 ÷ 2. If the algorithms are the same after removing parentheses, it does not count as originality; if the algorithms are different after removing parentheses, it counts as originality.

### Appendix D.3. Scoring Criteria for Question 3

(1) Fluency: One point is awarded for each correct answer (no points are given if the proof is incomplete or lacks key steps/auxiliary lines).
(2) Flexibility: One point is awarded for each category switch, specifically including:
  (i) C and D are on the same side of AB.
    ① Prove that △OCD ≌ △OBD
    ② Connect AD. In △ABE, the three lines are coincident (The median line, altitude and angle bisector on the base of an isosceles triangle coincide). Prove that △CDE ≌ △PDB
    ③ Prove that quadrilateral AODC is a rhombus, and then prove that quadrilateral OBDC is a rhombus
    ④ ∠BOD = ∠COD, BD = DC (Equal arcs imply equal chords)
    ⑤ ∠BOD = ∠COD, BD = DC (Equal angles imply equal chords)
    ⑥ By the cosine rule, DB^2^ = DC^2^, and the formula expansion shows that the angles are equal
    ⑦ OD is the median line, D is the midpoint, DC = DE (Angle chasing)
    ⑧ An exterior angle equals the opposite interior angle, ∠ECD = ∠OBD, equilateral, prove that △DCE ≌ △DBO
    ⑨ By the theorem of the median to the hypotenuse, prove that CD = 1/2 BE + median line
  (ii) C and D are on opposite sides of AB
    ⑩ Prove that △OCD ≌ △OBD
    ⑪ Prove that ∠COD = ∠BOD, equal angles imply equal chords
(3) Originality: Scored criteria same with Question 1.

Note: For the same type of answer from the same respondent, originality scoring is not repeated.

## Appendix E. MNI Coordinates of fNIRS Channel Midpoints

**Chanel**	**Name**	**x**	**y**	**z**
1	L Middle frontal gyrus	−51	23	46
2	L Middle frontal gyrus	−49	40	22
3	L Middle frontal gyrus	−42	39	44
4	L Middle frontal gyrus	−40	17	56
5	L Middle frontal gyrus	−26	34	56
6	L Supplementary motor area	−10	23	61
7	L Middle frontal gyrus	−42	58	17
8	L Middle frontal gyrus	−29	56	35
9	L Superior frontal gyrus	−14	62	21
10	L Superior frontal gyrus	−19	72	11
11	L Superior frontal gyrus	−14	48	53
12	L Medial superior frontal gyrus	−1	30	47
13	L Medial superior frontal gyrus	−1	47	32
14	R Superior frontal gyrus	15	46	53
15	R Medial superior frontal gyrus	1	57	12
16	L Superior frontal gyrus, orbital part	−17	68	−8
17	R Superior frontal gyrus, orbital part	16	69	−10
18	R Superior frontal gyrus	15	24	59
19	R Superior frontal gyrus	27	34	53
20	R Middle frontal gyrus	42	21	57
21	R Superior frontal gyrus	13	59	21
22	R Superior frontal gyrus	29	68	11
23	R Middle frontal gyrus	29	56	35
24	R Middle frontal gyrus	38	61	15
25	R Middle frontal gyrus	42	42	39
26	R Middle frontal gyrus	54	25	39
27	R Middle frontal gyrus	52	44	23
28	R Superior temporal gyrus	65	−12	−6
29	R Middle temporal gyrus	67	−29	−8
30	R Superior temporal gyrus	72	−22	7
31	R Superior temporal gyrus	62	3	6
32	R Supramarginal gyrus	62	−16	19
33	R Postcentral gyrus	64	2	34
34	R Middle temporal gyrus	68	−43	6
35	R Superior temporal gyrus	59	−29	19
36	R Angular gyrus	62	−50	38
37	R Middle temporal gyrus	62	−54	16
38	R Supramarginal gyrus	67	−24	40
39	R postcentral gyrus	60	−13	51
40	R Inferior parietal lobule	57	−32	53
41	R Superior parietal lobule	51	−32	62
42	R Inferior parietal lobule	50	−57	49
43	R Angular gyrus	60	−61	33
44	R Angular gyrus	41	−67	54
45	R Middle frontal gyrus	49	3	60
46	R Precentral gyrus	35	−10	66
47	R Superior frontal gyrus	22	4	72
48	R Superior parietal lobule	44	−47	63
49	R Superior parietal lobule	35	−61	66
50	R postcentral gyrus	35	−34	73
51	R Superior frontal gyrus	25	−46	79
52	R Precentral gyrus	26	−23	78
53	R Superior frontal gyrus	11	−8	77
54	R postcentral gyrus	11	−30	79

## Appendix F. Details of Data Analysis

1. Behavioral Data Analysis

About the scoring of Creativity tasks, including both general creativity and mathematical creativity, they were evaluated across three dimensions: fluency, flexibility, and originality. Fluency was scored by counting the number of correct responses, and if it is meaningful from mathematical perspectives, with one point awarded for each response. Flexibility was assessed based on shifts in problem-solving strategies; one point was assigned when two consecutive responses reflected different solution strategies. For example, in 24-point task, flexibility of the answer: “5 + 5 + 5 + 9, 5 × 4 + 2 + 2, 3 + 4 + 8 + 9, 4 × 8 − 5 − 3”, scored 3 points. Originality was evaluated using a statistical infrequency approach based on the distribution of responses across the full sample. Specifically, responses occurring in less than 1% of the sample were awarded 5 points; those occurring in 1.01–3% received 4 points; 3.01–5% received 3 points; 5.01–10% received 2 points; 10.01–15% received 1 point; and responses occurring in more than 15% of the sample received 0 points, with lower frequencies indicating higher originality.

All responses were scored by two raters, both doctoral students in mathematics education. Prior to formal scoring, the raters jointly reviewed a subset of responses to calibrate the scoring criteria. Because originality scoring depends on the distribution of responses across the complete sample, only fluency and flexibility were scored at the initial stage. Subsequently, responses were categorized according to problem-solving strategies relevant to flexibility, and all correct responses were compiled into a shared solution pool. During the scoring of the remaining responses, any discrepancies were resolved through discussion, and the solution pool and strategy classifications were revised accordingly. Through this iterative process, the scoring criteria gradually stabilized. Finally, originality scores were assigned based on the frequency of each response in the finalized solution pool. The intra-class correlation coefficient exceeded 0.87 for fluency, flexibility, and originality, indicating a high level of scoring reliability.

Based on the scoring results, independent-samples *t*-tests were used to assess baseline differences between the groups and to determine the significance of group differences following the priming intervention.

2. fNIRS Data Analysis

2.1. Preprocessing

Preprocessing was performed using the Homer3 toolbox and custom MATLAB (R2024a) scripts to ensure rigorous signal quality control. The pipeline included the following steps:

First, raw data were acquired at 25 Hz and down-sampled to 10 Hz to optimize the signal-to-noise ratio and computational efficiency. The signals were segmented by experimental stage, and raw light intensity was converted to optical density (OD). A sliding window procedure (10 s window) was used to identify artifacts, defined as data points exceeding ±3 standard deviations from the mean. Channels with over 5% of time points marked as artifacts were excluded and interpolated. Participants with over 30% of channels marked as bad were excluded from the analysis. This led to the removal of two participants. The average artifact percentage across all remaining participants and channels was 0.31%.

Subsequently, a wavelet-based motion correction algorithm was applied to the OD data. This method decomposes the signal into wavelet coefficients and removes outlier coefficients associated with rapid head movements. To mitigate systemic physiological interference, Principal Component Analysis (PCA) was employed; the first principal component, explaining >80% of the global signal variance, was regressed out from all channels. A bandpass filter (0.01–0.5 Hz) was then applied. The high-pass cutoff (0.01 Hz) removed slow instrumental drift, while the low-pass cutoff (0.5 Hz) attenuated high-frequency cardiac (~1 Hz) and respiratory (~0.3 Hz) noise. The filtered OD data were converted to oxygenated (HbO) and deoxygenated (HbR) hemoglobin concentrations using the Modified Beer-Lambert Law. HbO was selected as the primary metric for subsequent statistical analyses due to its higher signal-to-noise ratio and stronger correlation with the BOLD response. Finally, to normalize the signal variations relative to a common reference, a baseline correction was applied by subtracting the mean concentration of each channel calculated over the entire time course from its respective time series.

2.2. Priming Stage Analysis

To capture the temporal evolution of brain activity during the 20 min priming task, the data were segmented into 10 consecutive time windows of 60 s each. General Linear Model (GLM) was fitted to each window for every participant. The design matrix included a task regressor convolved with the canonical Hemodynamic Response Function (HRF). Beta values (*β*) derived from the GLM represented the activation intensity for each channel in each window. Independent samples *t*-tests were used to compare average activation across all windows and to analyze time-series activation differences.

To examine the relationship between neural activation and behavioral performance, we calculated Pearson correlation coefficients between the mean HbO activation and mathematical creativity scores for each channel. Correlation coefficients were converted to Z-scores using Fisher’s r-to-z transformation, and differences were assessed using independent sample Z-tests.

To capture temporal fluctuations in brain network organization, we calculated dynamic functional connectivity (dFC) using a non-overlapping sliding window approach, dividing participant time courses into ten 60 s discrete windows per block to generate 54 × 54 Pearson correlation matrices. Connection stability was quantified via Standard Deviation and Total Variation, with group differences assessed using independent samples *t*-tests. Recurring connectivity states were identified through k-means clustering, where the optimal cluster number (k) was determined by the Elbow method and validated via silhouette scores across 30 replications. State dynamics were characterized by Occupancy Frequency, Dwell Time, and Transition Probabilities; given the non-normal distribution of state metrics, group differences were evaluated using the Mann–Whitney U test, while transition probabilities were assessed using Fisher’s exact test (*p* < 0.05).

2.3. Post-Test Stage Analysis

Markers were used to define task blocks in post-test. Within these blocks, individual regressors were constructed for every valid solution provided by the participant. The onset time for each regressor was defined by the participant’s response marker, and the duration was calculated as the time interval to the subsequent marker (adjusted by a 2 s buffer). This trial-by-trial modeling allowed for the estimation of beta values for every unique solution generated. Subject-level activations were averaged across the three problem types to derive a mean activation map for the post-test stage. And group-level analysis was performed using channel-wise *t*-tests across 54 channels.

Brain-behavior correlations were analyzed to examine group differences between dimensions of mathematical creativity and neural activation. Moreover, to capture the multivariate patterns between brain activation and behavioral dimensions, we employed Partial Least Squares Correlation (PLSC). The neural data and behavioral data were input into a single model to identify latent variables that maximally explain the covariance between brain activity and behavior. The significance of the group effect and the generalizability of the LVs were tested using a permutation test with 5000 iterations. The model stability was evaluated by calculating the singular values and identifying the specific channel weights associated with the significant latent variables.

To examine network organization during problem-solving, weighted functional connectivity matrices (54 × 54) were constructed for each participant and problem block by calculating Pearson correlation coefficients between channel time series extracted from task intervals. Group differences in connection strength were evaluated utilizing independent samples *t*-tests on edge weights, with a strict Bonferroni correction applied to control for multiple comparisons across the 1431 network edges. Subsequently, network topological properties were analyzed by applying a proportional thresholding strategy (sparsity range: 0.05–0.40, step size: 0.05) to binarize the matrices. Global metrics (Global Efficiency, Characteristic Path Length) and local metrics (Clustering Coefficient, Local Efficiency) were computed at each threshold, and small-world topology was evaluated by normalizing these metrics against 100 degree-matched random networks to derive the small-world index. Finally, to mitigate threshold dependency, the area under the curve (AUC) was calculated for each metric, and group differences were assessed using independent samples *t*-tests with Cohen’s d used to quantify effect sizes.

## Appendix G. Analysis Results Table of Startup Stage Time

**Passage**	**Name**	**x**	**y**	**z**	**Time Window (t)**									
					**1**	**2**	**3**	**4**	**5**	**6**	**7**	**8**	**9**	**10**
1	L middle frontal gyrus	−51	23	46	0	0	0	0	0	2.03	0	0	0	0
2	L middle frontal gyrus	−49	40	22	0	0	0	0	0	0	0	0	0	0
3	L middle frontal gyrus	−42	39	44	0	0	0	0	0	2.59	0	0	0	0
4	L middle frontal gyrus	−40	17	56	0	0	0	0	0	0	0	0	0	0
5	L middle frontal gyrus	−26	34	56	−2.13	0	0	0	0	0	0	0	0	−2.4
6	L supplementary motor area	−10	23	61	0	0	0	0	0	0	0	0	0	0
7	L middle frontal gyrus	−42	58	17	0	0	2.03	0	0	0	0	0	0	0
8	L middle frontal gyrus	−29	56	35	0	0	0	0	0	0	0	0	2.05	0
9	L superior frontal gyrus	−14	62	21	0	0	0	0	0	0	0	0	2.32	0
10	L superior frontal gyrus	−19	72	11	0	0	0	0	0	0	0	0	0	0
11	L superior frontal gyrus	−14	48	53	0	0	0	0	0	0	0	0	0	0
12	L medial superior frontal gyrus	−1	30	47	0	0	0	0	0	0	0	0	0	0
13	L medial superior frontal gyrus	−1	47	32	0	0	0	0	0	0	0	0	0	2
14	R superior frontal gyrus	15	46	53	0	0	0	0	0	0	0	0	0	0
15	R medial superior frontal gyrus	1	57	12	0	0	0	0	0	0	0	0	0	0
16	L orbital part of superior frontal gyrus	−17	68	−8	0	0	0	0	0	0	0	0	0	0
17	R orbital part of superior frontal gyrus	16	69	−10	0	0	0	0	0	0	0	0	0	0
18	R superior frontal gyrus	15	24	59	0	0	0	0	2	0	0	0	0	2.67
19	R superior frontal gyrus	27	34	53	0	0	0	0	0	0	0	0	0	0
20	R middle frontal gyrus	42	21	57	0	0	0	0	0	0	0	0	0	0
21	R superior frontal gyrus	13	59	21	0	0	0	0	0	0	0	0	2.05	0
22	R superior frontal gyrus	29	68	11	0	0	0	0	0	0	0	0	0	0
23	R middle frontal gyrus	29	56	35	0	0	0	0	0	0	0	0	0	0
24	R middle frontal gyrus	38	61	15	0	0	0	2.52	0	0	0	0	0	0
25	R middle frontal gyrus	42	42	39	0	0	0	0	0	0	0	0	0	0
26	R middle frontal gyrus	54	25	39	0	0	0	0	0	0	0	0	0	0
27	R middle frontal gyrus	52	44	23	0	0	0	0	0	0	0	0	0	−2.11
28	R superior temporal gyrus	65	−12	−6	0	0	0	0	0	0	0	0	0	0
29	R middle temporal gyrus	67	−29	−8	0	0	0	0	0	0	0	0	0	0
30	R superior temporal gyrus	72	−22	7	0	0	0	0	0	2	0	0	2.28	0
31	R superior temporal gyrus	62	3	6	0	0	0	0	0	0	0	0	0	0
32	R superior parietal gyrus	62	−16	19	−2	0	0	0	0	0	0	0	0	0
33	R postcentral gyrus	64	2	34	0	0	0	0	0	0	0	0	0	0
34	R middle temporal gyrus	68	−43	6	0	0	0	0	0	0	0	0	0	0
35	R superior temporal gyrus	59	−29	19	0	0	0	0	−2.12	0	0	0	0	0
36	R angular gyrus	62	−50	38	0	0	0	0	0	0	0	0	0	0
37	R middle temporal gyrus	62	−54	16	0	0	0	0	0	0	0	0	0	−2.14
38	R superior parietal gyrus	67	−24	40	2.63	0	0	0	0	2.56	0	0	0	0
39	R postcentral gyrus	60	−13	51	0	0	0	0	0	0	0	0	2.74	0
40	R inferior parietal lobule	57	−32	53	0	0	0	0	0	0	2.64	0	0	0
41	R superior parietal lobule	51	−32	62	0	0	0	0	0	0	0	0	0	0
42	R inferior parietal lobule	50	−57	49	0	−2.16	0	0	0	0	0	−2.72	0	0
43	R angular gyrus	60	−61	33	0	0	0	0	2.29	0	0	0	0	0
44	R angular gyrus	41	−67	54	0	0	0	2.51	0	0	0	0	0	0
45	R middle frontal gyrus	49	3	60	0	0	0	0	0	0	0	0	0	0
46	R precentral gyrus	35	−10	66	0	0	0	0	0	0	0	0	0	0
47	R superior frontal gyrus	22	4	72	0	0	0	0	0	0	0	0	0	0
48	R superior parietal lobule	44	−47	63	0	0	0	0	0	0	0	0	2.41	0
49	R superior parietal lobule	35	−61	66	0	0	0	0	0	0	0	0	2.51	0
50	R postcentral gyrus	35	−34	73	2.2	0	0	0	0	0	0	−2.24	2.03	0
51	R superior parietal lobule	25	−46	79	0	0	0	0	0	0	0	−2.54	2.61	0
52	R precentral gyrus	26	−23	78	0	0	0	0	0	0	0	0	2.16	−2.24
53	R superior frontal gyrus	11	−8	77	0	0	0	0	0	0	0	0	0	0
54	R postcentral gyrus	11	−30	79	0	0	0	0	−2.06	0	0	0	2.14	0

## Appendix H. PLSC Analysis Results of Brain-Behavior Correlation During the Post-Test Stage

A total of 1 latent variable (Latent Variable, LV1) was extracted through the analysis. Permutation test results showed that the inter-group effect size of this latent variable was 1.66, with a permutation test *p*-value of 0.07 (*p* > 0.05), indicating a marginally significant difference. This suggests that there is a moderate trend of differentiation in the brain-behavior correlation patterns between the two groups of participants (see Figure A1, top-left).

Further analysis of the regression relationship between brain activation and composite behavioral scores in the two groups revealed: the composite behavioral scores of the auxiliary line group had a better predictive effect on brain activation (regression equation: y = 37.22x + 2.33); the control group showing relatively weaker predictive power (regression equation: y = 26.58x − 2.92) (see Figure A1, top-right).

Behavioral weight analysis indicated that the current latent variable could explain 22.26% of the behavioral variance. Among them, the behavioral weights of the originality and fluency dimensions were relatively higher, which is consistent with the behavioral experimental results (the auxiliary line group was significantly superior to the control group in these two dimensions) (see Figure A1, bottom-left).

Brain region weight analysis showed that the latent variable could explain 7.15% of the brain activation variance. The Right Precentral Gyrus and Right Superior Frontal Gyrus exhibited strong positive weights, indicating that the correlation between these brain regions and behavioral indicators was the most significant (see Figure A1, bottom-right and Table A1). Information on the 5 channels with the largest brain weights in the PLSC analysis is shown in Table A1.

**Table A1:** Information on the 5 channels with the largest brain weights.

Channel	Brain Region	x	y	z	w
52	R Precentral gyrus	26	−23	78	3.64
19	R Superior Frontal Gyrus	27	34	53	2.90
14	R Superior Frontal Gyrus	15	46	53	2.39
18	R Superior Frontal Gyrus	15	24	59	2.09
31	R Superior Temporal Gyrus	62	3	6	−1.82

## Appendix I. Correlation Between Priming Stage and Post-Test Stage

The correlation between brain activation in the two stages of the auxiliary line group was significantly higher than that of the control group (*p* < 0.05) in the following brain regions: Right Medial Superior Frontal Gyrus, Right Orbital Part of Superior Frontal Gyrus, Right Superior Frontal Gyrus, Right Middle Frontal Gyrus, Right Superior Temporal Gyrus, Right Inferior Parietal Lobule, and Right Precentral Gyrus.

The correlation between brain activation in the two stages of the control group was significantly higher than that of the auxiliary line group (*p* < 0.05) in the following brain regions: Left Middle Frontal Gyrus, Left Orbital Part of Superior Frontal Gyrus, Right Postcentral Gyrus, and Right Angular Gyrus. This indicates that the brain activation in the priming stage of the control group had a stronger predictive power for brain activation in the mathematical creative thinking task in the post-test stage (see Figure A2 and Table A2).

**Table A2:** Coordinates and statistical parametric of difference in correlation between priming stage and post-test brain activation.

Channel	Brain Region	x	y	z	r1	r2	z
4	L Middle Frontal Gyrus	−40	17	56	−0.46	0.62	−2.38
5	L Middle Frontal Gyrus	−26	34	56	−0.20	0.66	−3.02
15	R Medial superior frontal gyrus	1	57	12	0.89	0.42	2.97
16	R Orbital part of Superior Frontal Gyrus	−17	68	−8	−0.67	−0.04	−2.31
17	R Orbital part of Superior Frontal Gyrus	16	69	−10	0.06	−0.90	4.62
22	R Superior Frontal Gyrus	29	68	11	0.51	−0.60	3.78
24	R Middle Frontal Gyrus	38	61	15	0.68	−0.08	2.72
27	R Middle Frontal Gyrus	52	44	23	−0.17	−0.71	2.17
28	R Superior Temporal Gyrus	65	−12	−6	0.43	−0.88	5.56
31	R Superior Temporal Gyrus	62	3	6	0.09	−0.73	3.08
36	R Angular gyrus	62	−50	38	0.42	−0.46	2.82
39	R Postcentral gyrus	60	−13	51	−0.22	0.45	−2.13
44	R Angular gyrus	41	−67	54	−0.14	0.82	−3.87
46	R Precentral gyrus	35	−10	66	0.04	−0.59	2.18
54	R Postcentral gyrus	11	−30	79	−0.32	0.33	−2.05
